# Assessing Serum Brain Derived Neurotrophic Factor and Matrix MetalloProteinase-9 Levels and Their Correlation With Neurocognitive and Psychosocial Functioning in Bipolar Disorder-I in Remission: A Case-Control Study

**DOI:** 10.1192/bjo.2022.233

**Published:** 2022-06-20

**Authors:** Venkatalakshmi Penchilaiya, Shivanand Kattimani, Nandheesha Hanumanthappa, Arivazhagan Karunanithi

**Affiliations:** 1 Northamptonshire Healthcare NHS Foundation Trust, Northampton, United Kingdom; 2 JIPMER, Pondicherry, India

## Abstract

**Aims:**

1. To determine the association between serum BDNF, serum MMP-9 and cognitive function test in BD-1 patients in remission and to compare with controls.
2. To assess the current psychosocial functioning of BD-I patients in remission and their correlates.

**Methods:**

Single center case control study.

Cases were BD-I patients in Remission (**n = 60**) and controls (**n = 60**) were age and gender matched healthy persons. The diagnosis of BD-1 was confirmed using **Structured Clinical Interview For DSM-IV-Tr Axis I Disorders –Research Version** along with clinical record. Age group between**18–60 years**, in **remission for at least 2 months** [scoring ≤ 8 on the Hamilton Depression Rating Scale, and ≤ 6 on Young Mania Rating Scale] were included. Those with significant head injury, neurological disorder, substance use disorder, Diabetes/Hypertension and premorbid IQ < 70 were excluded.

Control group were excluded if their first degree relative had any psychiatric illness as elicited using Family Interview for Genetic Studies scale (**FIGS)**.

Cognitive functioning was assessed using Addenbrooke's Cognitive Examination version III (**ACE-III)** and Trail making test A and B (**TMT A and TMT B**). Current psychosocial functioning was assessed with Functioning assessment short test (FAST).

Five ml blood sample was taken for estimation of serum BDNF and MMP-9 levels by ELISA.

**Chi-square** test used to compare categorical variables. **Mann-Whitney U test** for continuous variables. **Spearman's correlation** - evaluate the relationship between scores on the cognitive function tests and serum levels of BDNF and MMP-9, within the group of patients with BD-I.

**Results:**

With regards to cognitive functioning, compared to controls, cases performed significantly poor in domains of **Memory** (Z = −3.435, p = 0.001), **Processing speed** (z=−2.667, p = 0.008), and E**xecutive functioning** (Z= −4.084, p = 0.000).

**No statistical difference in levels of serum BDNF and MMP-9** between patients and controls were found.

While BDNF serum levels were not associated with cognitive or psychosocial functioning, there were significant relation between **serum MMP-9 and the various domains of FAST scale and total FAST score (rho = 0.447, p < 0.001).**

BD-I patients exhibited **poor psychosocial functioning** compared to controls even in euthymic state (U = 702.00, p < 0.000).

**Conclusion:**

Patients with BD-I display **poor performance in memory, executive function and psychosocial functioning** even during euthymic state compared to controls.

Serum BDNF and MMP-9 levels comparable to the healthy controls during remission- pointing towards them as **state markers** rather than trait.

Need for **routine evaluation of cognitive functio**n during follow-up visits and f**ocus on target deficits for rehabilitation** for better recovery and improving the quality of life of BD patients.

